# Breast Cancer: The Psychological Impact of Diagnosis, Treatment, and Remission

**DOI:** 10.7759/cureus.70814

**Published:** 2024-10-04

**Authors:** Joseph Graham

**Affiliations:** 1 Neurosurgery, Royal Sussex County Hospital, Brighton, GBR

**Keywords:** body dissatisfaction, breast cancer management, clinical anxiety, depression, depression in medical disease, patient-centred care, psycho-oncology

## Abstract

Breast cancer affects millions of people worldwide. With physical manifestations being the predominant feature of management, healthcare professionals can overlook the psychological toll that the disease can have on the patients and their support network. This literature review examines the vast multi-factorial approach that must be taken when managing breast cancer patients from initial screening to diagnostic investigations, treatment, and remission. A literature search in PubMed from January 2000 to April 2024 was executed. Data sets in the studies filtered during the literature search were collected and analysed, looking not only at the data itself but also the entirety of the study. This included its limitations and possible biases. From screening, the possibility of cancer as a diagnosis can trigger mixed emotions including fear, depression, and anxiety. During diagnostic, patients may find themselves subject to fear of negative body image evaluation and fear of judgment. Medical professionals must be prepared to support the patient when they experience these feelings. The treatment stages can be the most difficult for the patient as side effects and complications of treatment can impact their lives in numerous ways, making management challenging. These include pain, sexual dysfunction, and alopecia.

Overall, the analysis of the selected literature showed areas in clinical practice that can be optimised when providing psychological support for a patient’s cancer diagnosis, management, and treatment. Being able to counsel prior to the presentation of these, and ideally prevent unnecessary cases of these can substantially increase a patient’s quality of life during treatment. This literature review hopes to identify and promote awareness and further implementation of support systems by healthcare professionals.

## Introduction and background

Breast cancer is the most commonly diagnosed cancer worldwide with a total of 2.26 million newly diagnosed cases in 2020. Breast cancer subsequently has the highest number of deaths of all cancers with 684,996 deaths in 2020 [1]. In the UK, one in every seven women will be diagnosed with breast cancer during their life, proving nationally to be the most prevalent cancer. Among men, 370 are diagnosed with breast cancer every year, a notable difference, however still present [2].

Breast cancer patients will suffer from numerous clinical features throughout the course of their illness. This can be divided into the patient’s symptoms prior to presentation, investigation, and diagnosis, and then during the treatment stage where the patient might be undergoing single or multiple interventions. Finally, when patients have successful treatment, they will be followed up as surveillance cases. Overall, the biopsychosocial model by Engel (1977) can be applied when performing an analysis [3]. This model incorporates three domains of approaching healthcare and individual patient issues, dividing it into a biological section, a psychological section, and a social domain. Regarding treating and managing breast cancer patients, the psychological and social aspects must always be considered. The emphasis of treatment is however placed on the biological or medical aspect, as these are deemed appropriately as the most life-threatening.

The commonest symptoms of breast cancer can be categorised into "typical" and "atypical" presentations. Typical symptoms refer to the presence of a breast lump, and the symptoms associated with the presence of a mass. Atypical symptomatic presentations refer to patients attending with features other than that of a breast lump. These include nipple abnormalities, breast pain, skin changes such as a peau d'orange or erythema, and axillary lumps [4]. This narrative review incorporated data from January 2000 to April 2024. A search on PubMed was performed to identify any relevant studies to the topics described. This included keywords such as "breast cancer", "psychology", "anxiety", "fear", and "depression", as well as the accordant section such as diagnosis, treatment, and remission. The data itself was then analysed, along with the entirety of the study. Looking at the limitations and strengths of the studies, in addition to the possible biases.

The psychological status of the patient must be addressed by the Multi-Disciplinary Team (MDT) during treatment. Various breast cancer charities such as the Breast Cancer Now support group, in association with NICE (National Institute for Health and Care Excellence) guidelines provide assistance and support groups for patients at different stages in their cancer journey [5]. The healthcare professionals can then provide the patient and their support network with resources, as opposed to their actively seeking it out. A meta-analysis by İzci et al. (2016) collected the most frequent mental health disorders experienced by breast cancer patients, with depression and anxiety being experienced by an average of 31.7% of patients [6]. The aim of this narrative review is to broadly analyse the psychological effects that the different stages of the cancer pathway have on a patient. This has been divided into three sections, looking at diagnosis initially, then treatment, and finally remission. During these stages, the findings of the studies have been described including the limitations. Overall, the goal is to identify the regions of improvement that healthcare professionals can implement on an individual, local and national level in order to decrease the negative psychological findings described.

## Review

Impact of diagnosis on patient’s psychological health

Breast Screening Programmes

In 1988, the National Health Service Breast Screening Programme (NHSBSP) was introduced to the general population [7]. The NHSBSP invited all women aged 50 to 70 years for a mammogram every three years [7]. Following the mammogram, the result is communicated to the patient within two weeks. If the scans reveal a suspicious lesion or there was a technical error, the woman will have an appointment arranged within three weeks of the initial scan. This ‘after screening test’ may include repeating the clinical examination, performing an ultrasound, re-performing mammography, or a biopsy of the lesion/s [8]. The results of these further investigations will be discussed in the MDT meeting, where the appropriate further investigation, management and treatment will be decided. All of this will then be communicated and discussed with the patient. The MDT meeting itself may include the parent team of the patient, the oncologists, the breast surgeons, specialised breast care nurses, histopathologists, radiologists, and occupational and physiotherapists. 

Screening Programmes

During the screening and diagnosis of possible breast cancer, the majority of the emphasis of the patient’s care is on the biological segment of the biopsychosocial model. The levels of anxiety experienced by a patient vary depending on whether this is the individual’s first mammogram screening or if this was a return visit for additional investigation post-biopsy. Lourenco and Baird (2017) investigated the effect of community education on this anxiety, using a self-report anxiety scale of 0-10, where 0 is no related anxiety and 10 is "extreme" [9]. This study showed that patients reported an average of 3.6/10 on a self-reported anxiety scale when presenting for a routine mammogram, whereas when being invited for additional investigative screening, anxiety rose to 8.0/10. This level was similar for patients requiring biopsy for diagnosis, 8.6/10. Limitations for this can be identified for this specific as there was a small sample size of only 27 women, and only a single relative scale was used for comparison. The aim of this study was to identify if there were changes in anxiety, post-education on why the investigations were being performed. This did show an improvement in pre- and post-knowledge, thereby emphasising the importance of public education on the topic [9]. In relation to the NHSBSP, the question of public education surrounding breast cancer can be posed. Does a lack of education introduce additional barriers to screening and diagnosis? A limitation of analysing this study was the inability to access the complete self-reported anxiety scale questionnaire that was used. They do describe the questions in the form of "anxiety associated with mammography, call back, and biopsy", however not the actual questions themselves. 

Lee et al. (2015) analysed more variables regarding the cause of this increased screening-associated anxiety [10]. These included minimizing waiting times, improved communication between patients and breast centre staff, relaxation techniques and a professional environment. The study had a near consensus that if the results of the investigations were immediate, then anxiety would be reduced, even as significant as a 75% relative decrease [10]. Barentsz et al. (2014) further studied the impact of immediate core-needle biopsy if indicated by imaging on the patient’s anxiety level [11]. With no decrease in the accuracy of the resulting diagnosis, the patient’s anxiety decreased, thereby supporting the findings of Lee et al. (2015) [10]. Ideally, this would occur in all breast screening clinics, however, due to numerous staffing and funding barriers, this is not always feasible. The study also investigated the different environments that individuals would find more relaxing and less stress-inducing. These include providing distractions for patients such as a variety of entertainment in the waiting rooms, and "spa-like atmospheres" to support this. A collection of these has now been implemented in numerous settings and clinics in order to contribute to supporting the patient in reducing anxiety.

Intimate Examinations

The concept of intimate examinations acts as a barrier for women to attend screening appointments, as well as extending out to impeding self-examination in the absence of a healthcare professional. Consedine et al. (2004) investigated the barrier of “anxiety, fear, and worry” regarding breast cancer screening [12]. The meta-analysis performed identified three sources of anxiety in patients’ apprehension of attending screening appointments: “(a) fear of screening components, (b) fear of screening outcomes, and (c) undifferentiated cancer fear” [12]. Undifferentiated cancer fear is a term used in the meta-analysis that refers to an "undifferentiated fear or anxiety of getting cancer". These are not mutually exclusive but have significant overlap. Studies included in the meta-analysis found specific worries including pain associated with the imaging, fear of complications of the procedures, fear of the outcome, and embarrassment of the procedure itself [12]. Sharif et al. (2020) analysed the concept of embarrassment further, recognising it as a barrier to screening [13]. Fear of negative appearance evaluation (FNAE) as an obstacle to attending, refers to an individual’s concern about “negative interpersonal evaluation”, thereby resulting in avoiding the scenario as a whole [13]. Among women, 66.4% self-reported that FNAE influenced them to avoid or change the location of screening [14]. With consideration of a limited sample size and a possibly biased demographic, this significant statistic requires consideration by healthcare professionals at the local scale, and governments on a national scale. Modifications have been made, as mentioned previously, to empower patients and decrease levels of worry and anxiety associated with screening, such as providing a private place to change into gowns and allowing for the patient to decide whether a healthcare chaperone be present.

Symptom Presentation

Maheu et al. (2019) studied the anxiety and uncertainty of women presenting to Rapid Diagnostic Clinics (RDCs) with suspicious symptoms [15]. Using qualitative analysis techniques, the anxiety and stress of the patients was measured pre- and post-diagnosis. 13 women were recruited for the study, who presented to the breast clinic with suspicious signs and/or symptoms. The participants were then subdivided pre-diagnostically depending on whether they were assessed as having high uncertainty but low anxiety levels or both high uncertainty and anxiety levels. Interviewing the former group, Maheu et al. (2019) concluded that two main themes arose [15]. The initial being that they saw the scenario as positive and optimistic, that the outcome would be that the lesion was benign. The women described using “repetitive positive thoughts” in order to reassure themselves and conclusively reduce their anxiety levels. The second theme includes the support and reassurance received from the healthcare staff working in the RDCs. When debriefing with the latter group with high levels of anxiety and uncertainty, the theme of “maintaining responsibilities” continually arose in the various patients. Recurrently, participants described that the possibility of a cancer diagnosis would drastically change their quality of life and activities of daily living. This therefore ruminated in this group of patients, ultimately resulting in anxiety and uncertainty levels rising. Patient repeatedly specified the subject of their children and how their ability “to be a good mother” would be affected, and how their “future, as a mother” would be impacted negatively. This impact would be from a multitude of different factors including the psychological burden of suffering from cancer as well as the physical and mental toll that the various treatments can have [15].

Impact of treatment on patient’s psychological health

Chemotherapy

The grading and severity of the breast cancer will determine what specific chemotherapy regimen a patient undergoes. Zanuso et al. (2020) analysed the relationship between the side effects of adjuvant chemotherapy and outcomes in elderly breast cancer patients and identified the most frequently experienced signs and symptoms [16]. These include most commonly nausea and vomiting, diarrhoea, mucositis, and signs of infection secondary to a depleted white cell count [16]. The effect of these clinical features can be detrimental to not only a patient’s quality of life but also that of their relatives and supporting network. Patients also experience anxiety and fear prior to commencing chemotherapy treatment, as they may have previous experiences, direct or indirect, of the effects.

Hwang et al. (2013) took a broad approach to assess how and to what extent adjuvant chemotherapy for breast cancer affects a patient’s quality of life (QOL) [17]. They analysed four categories with different questionnaires being answered by the patients. These sections include the Functional Assessment of Cancer Therapy-Breast (FACT-B), the Ladder of Life single-item measure, the sexuality section of the supportive care needs survey (SCNS) and the Beck Depression Inventory (BDI) [18-21]. The FACT-B questionnaire includes a 36-item questionnaire aimed at assessing the general effect of cancer and chemotherapy on their QOL, with specific questions associated with breast cancer [18]. The ladder of life provides a rough summary of a patient’s impressions on their QOL by asking on a scale of one to ten, with one being the worst possible life and ten being the best possible life, where they would position themselves at that present moment. The SCNS is a survey established by McElduff et al. in 2004 and provides five domains in which patients answer on a grade of not applicable to high need [20]. The sexuality needs section allows the researchers to assess a patient’s sexual feelings and how this relates to their relationships (Table 1). Finally, the BDI (version II) is a common questionnaire used when assessing depression and allows for the quantification of the patient’s symptoms and attitudes [21]. A BDI score of 0-13 indicates none or minimum depression, 14-19 as mild depression, 20-28 as moderate, and 29 and above as severe, and is scored out of a total of 63.

**Table 1:** Sexuality needs domain of Supportive Care Needs Survey (SCNS). [20] This sexuality domain of the SCNS explores a patient’s change in sexual feelings, any changes in sexual relationships and any information about sexual relationships. Researchers are then able to evaluate the impact the illness is having on not only the patients themselves but their partners. The total SCNS consists of 34 questions, however these three relate to the sexual needs of the patient.

In the last month, what was your level of need for help with:	No need		Some need		
	Not applicable	Satisfied	Low need	Moderate need	High need
Changes in sexual feelings	1	2	3	4	5
Changes in your sexual relationships	1	2	3	4	5
To be given information about sexual relationships	1	2	3	4	5

**Figure 1:**
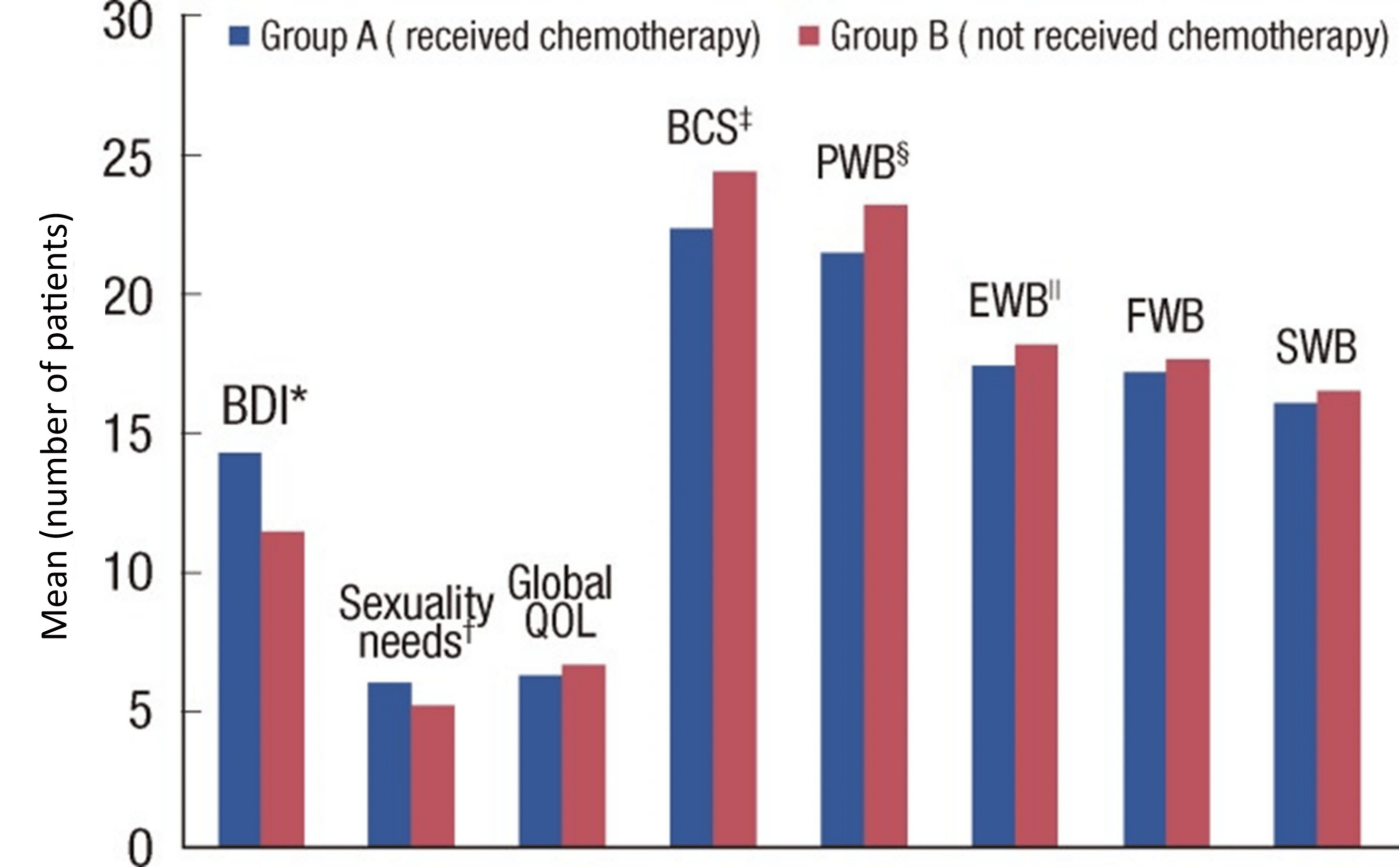
Comparison of aspects of quality of life between breast cancer patients undergoing chemotherapy and those not receiving chemotherapy. This diagram is a bar chart representation comparing the different aspects of quality of life (QOL) and overall mental health in patients who received chemotherapy (Group A) and those who did not receive chemotherapy (Group B). Patients who underwent chemotherapy had higher depression and sexuality needs scores, corresponding to increased levels of depression and sexual dysfunction. The remaining categories including breast cancer subscale, physical well-being, emotional well-being, functional well-being and social well-being were all also higher in the non-chemotherapy group. This indicates a statistically significant greater QOL in patients, including the BCS, PWB, EWB, FWB, and SWB subsections, not undergoing chemotherapy. BDI: Beck Depression Inventory, QOL: quality of life, BCS: breast cancer subscale, PWB: physical well-being, EWB: emotional well-being, FWB: functional well-being, SWB: social well-being.

By using these distinct questionnaires and surveys, Hwang et al. could statistically analyse the effect of chemotherapy on patient’s quality of life and their overall mental health, with the results portrayed in a bar chart (Figure 1) [17]. The conclusions were that depression and sexuality needs for patients undergoing chemotherapy were higher than those not receiving chemotherapy, however the FACT-B questionnaire results were greater in patients not undergoing chemotherapy [18]. The results for the FACT-B questionnaire were significantly high in both groups, despite it being higher in non-chemotherapy-treated patients. We can still acknowledge the considerable effect of chemotherapy on the patient’s QOL. Hwang et al. were able to incorporate a wide range of sections which all contribute to affecting a patients QOL, seeing the affect chemotherapy has on them [17]. Using the different sections, we can gain insight into the significant and possibly detrimental effects cancer treatment can have on individuals and their partners. These provide a psycho-oncological perspective into how in the future, healthcare professionals can screen for and provide treatment for patients in need [17].

Radiotherapy

Radiotherapy is frequently used in breast cancer adjuvant treatment as it significantly decreases the risk of local recurrence. With adequate radiotherapy and positive surgical margins, radiotherapy can decrease rates of regional relapse rates by up to 90% and decrease distant metastasis in patients with triple-negative breast tumours [22]. Silva et al. (2022) approached the investigation regarding the QOL of breast cancer patients undergoing radiotherapy in a similar manner to Hwang et al. [17,23]. A questionnaire developed by the European Organisation for Research and Treatment of Cancer known as the Quality of Life of Cancer Patients (QLQ-C30) was provided to a small sample size of women between their sessions of radiotherapy [23]. The results found that the highest percentage change between session one (day one) and session 28 (day 28) was related to the time needed to rest, sleeping disturbance, generalised weakness, and fatigue. The authors calculated a patient’s mean symptom score on day one to be 13.85, and then on day 28 to be 24.62. This study also investigated the patient’s differences in self-image and sexual satisfaction as with Hwang et al. [17]. Corroboration with other studies including Sharma and Purkayastha (2017), showed that individual’s sexual activity, sexual satisfaction, sexual function, and body image decreased throughout the duration of their radiotherapy treatment [24]. Limitations of this comparison are the inclusion criteria of the groups of patients in the study. Sharma and Purkayastha (2017) specifically interviewed postmastectomy carcinoma breast patients, whereas Silva et al. had 70% of patients having undergone chemotherapy cycles and 30% having had a form of surgery [23]. With these two studies, there is a larger sample size, including a wider scale of patients who have experienced different primary treatments with the use of adjuvant radiotherapy, showing an overall increase in symptom presence and a general decrease in QOL over the sessions recommended. 

Symptom Effect on Quality of Life

During a patient’s cancer pathway, they will experience a myriad of different symptoms affecting one or more systems. These may be classified as "organic" or "non-organic" symptoms. Organic refers to the being direct caused by the presence of the cancer and or spread, whereas non-organic or functional symptoms mean that they are not explained by the disease. Aranda et al. (2005) found that metastatic breast cancer patients reported an average of 14 symptoms, with a decline in sexual function being the most frequent [25]. The most distressing symptom was noted to be pain [25]. Having observed the effect chemotherapy and radiotherapy have on QOL, symptomatic management for patient complaints is necessary to not only attempt to increase it but to prevent further decline.

Pain

In reference to breast cancer, 71.7% of patients in a cross-sectional study reported pain which correlated with an overall decrease in QOL [26]. Greco et al. (2014) concluded that 43.4% of patients with cancer had undertreated pain, measured through the Pain Management Index (PMI) [27]. However, since 1994, there has been a substantial decrease in 32% of patients in a systematic review reporting undertreatment of pain. The study acknowledges the difficulty of not only treating the different types of pain but initially diagnosing them. There is also consideration of external factors that result in undertreatment of pain including social-economic and geographic trends revealing that higher GDP nations, such as Germany, France and the United Kingdom, have higher rates of cancer pain management [27]. This reinforces the importance of the bio-psychosocial model when treating cancer patients, as multifactorial approaches to understanding patient’s situations must be considered whenever possible. 

Sexual Dysfunction

Sexual dysfunction was the most frequent symptom reported by Aranda et al. (2005) [25]. Breast cancer patients describe vaginal dryness and dyspareunia as frequent symptoms and can be classified under the genitourinary syndrome of menopause (GSM). These symptoms are commonly associated with the use of aromatase inhibitors and selective oestrogen receptor modulators (SERMs), such as anastrozole and tamoxifen respectively, in hormone-sensitive pre-menopausal breast cancer patients. These drugs can mimic the underlying physiology of menopause, including the oestrogen-deficient state and surge in gonadotropins. This then leads to the main problems of “penetration pain, lubrication, dysfunctional desire and dysfunctional excitement” [28]. The categories of breast cancer sexual dysfunction can be classified depending on the predominant cause, physiological and psychological. Physiological refers to the hormone disturbances triggered by medications and psychological can relate to body image distortions, hormone effects on libido, and effects on the patient’s relationship with their partner. Boswell and Dizon (2015) explored the intervention options in an attempt to treat this [29]. Topical testosterone showed decreased rates of dryness and dyspareunia, with similar results with vaginal dehydroepiandrosterone, aqueous lidocaine, vaginal gel, and vaginal moisturiser [29]. Due to the high volume of literature released regarding complaints of sexual dysfunction during and post-treatment, oncologists are able to screen for these signs and symptoms earlier on in treatment and even ideally prevent them from occurring.

Chemotherapy-Induced Alopecia

Hair loss during chemotherapy, also known as chemotherapy-induced alopecia (CIA) can be a distressing side effect of certain agents. Hair loss leads to a distortion of the patient’s appearance and therefore can impact their QOL concerning aspects such as self-esteem, body image, and sexual and social interactions. A meta-analysis was performed collecting the results from numerous studies concerning a patient’s impressions and feelings toward the possibility of hair loss and or their emotions after the hair loss has occurred. The surveys showed that chemotherapy-induced alopecia commonly ranks in the top troublesome adverse effects due to the secondary impacts. Reports have even shown a sample of 8% of women contemplated refusing chemotherapy regimens as a whole due to the risk of induced alopecia. During qualitative studies, a repeating idea that was mentioned was the concept of the loss of privacy of the patients. This refers to other people noticing that you are undergoing chemotherapy treatment, likewise, it is a remembrance that a patient is currently in treatment. There was also an acknowledgement that this may have negatively impacted patients who wanted to return to work or continue part-time during the treatment [30].

Lemieux et al. (2007) performed a meta-analysis where the re-occurring themes were collected and divided into qualitative and quantitative studies [31]. These concern ideas of privacy, sexuality changes and professional effects mentioned. Other themes included attempting to conform and “be normal”, trying to take control of their disease and side effects, and constantly being a reminder that they were fighting cancer. From a quantitative perspective, women less than 50 years old had a consequential decrease in body image due to hair loss. There is however some dispute amongst results within studies, showing that the effect on body image cannot be directly correlated with hair loss, but is vastly multifactorial with the other bio-psychosocial aspects of cancer and its therapies [31].

Solutions

Solutions to tackle anxiety related to hair loss in cancer treatment have been researched and implemented in modern day oncology. The “Look Good, Feel Better” programme was created in 1989 in the UK to support not only breast cancer patients, but anyone suffering from emotional or confidence issues relating to their appearance [32]. Wig programs and Appearance-Care Programmes (ACPs) play a role in this charity to promote self-management and allow patients to regain control of their illness and their appearance [32].

Palliative Therapy

Palliative therapy for advanced breast cancer patients is a complicated and multi-disciplinary approach as each patient is different. As seen, associations and complications of breast cancer are not limited to the physical manifestations and infiltration of the tumour itself. Plans are therefore formulated to focus on the various parties involved, the patient, the relations, and the healthcare professionals. Each member of the team must be aware of the goals of treatment and the respective limitations, with regular meetings to evaluate progress. Guidelines have been published relating to the numerous presenting complaints that patients can present with, including both physiological and psychological. Focussing on the psychological, Cherny et al. (2018) evaluated that anxiety and depression frequently present concomitantly [33]. The notable presentations include themes of fearfulness, thought confusion, restlessness, uncertainty, and fear of progression. The combination of pharmacological and non-pharmacological treatment is used, with the mainstay of pharmacotherapy being anxiolytics. If resistant, atypical antipsychotics are indicated. In regard to non-pharmacological therapy, supportive psychotherapy and other behavioural interventions can be employed in order to tackle some of the issues that arise throughout the cancer journey. The study includes examples of these goals; decreasing the sense of isolation, motivating a patient’s self-confidence and respect, and analysing the emotions of being unsure regarding the length and quality of their future. Approaching these issues can be challenging for the healthcare professionals involved, and if screening techniques are not performed, this may obstruct the patient from ultimately achieving the necessary pharmacological and or non-pharmacological therapy [33].

Rodin et al. (2018), researched the impact of Cancer and Living Meaningfully (CALM) intervention on patients with advanced cancer [34]. CALM is a new intervention that aims to prevent the negative psychological impacts that advanced cancer can have on individuals. The study looked at the difference in depressive symptoms in patients who underwent CALM participation compared the the usual care. CALM therapy incorporated concepts such as providing patients with "safe places" to talk about the issues that they have been and therefore allowing them to "grow as a person". Overall the results showed no adverse effects when undergoing the novel therapy and also showed a significant increase in end-of-life preparation at 6 months compared to the usual interventions made. Further studies into CALM therapy for breast cancer are required to further identify the needs and requirements for specifically breast cancer patients [34]. 

Impact of remission on patient’s psychological health

Mastectomy

Over the years, various mastectomy techniques and procedures have been introduced and implemented, however, the overall aim remains the same. Individuals’ perception of the mastectomy will depend on their previous knowledge and experience with the procedure. Thakur et al. (2022) reported substantial rates of disturbance in body image, with 92% of the women post-mastectomy scoring significant distress on the 10-item Body Image Scale [35,36]. An important note from the study quoted is that the psychological impacts and disorders in body image perception remain significant issues that require attention even after the completion of therapy. Comparing new survivors (< one year of completion of treatment) and old survivors (> one year of completion of treatment), levels of depression and negative body image scores decreased (Table 2). Reasons underlying this decrease in rates of depression suggested by the study include adaptation to the stresses introduced by breast cancer and decreased economic demand due to the cessation of treatment. 31.5% of the study population were identified as having anxiety, interestingly showing no statistically significant change from the time of completion of treatment. The improvements and future implementations suggested by Thakur et al. were that oncological settings need to be prepared with healthcare professionals and psychological aids [35].

**Table 2:** Comparison of depression anxiety, stress and body image scores in new and old survivors of breast cancer. [35] This table shows the difference in levels of depression, anxiety, stress and body image score between new survivors (within 12 months of the completion and their prescribed treatment) and old survivors (greater than a year since treatment completion). Analysing the table we can see that that all the variables mentioned are lowed in old survivors group, however only depression and body image score are statisically significant (p < 0.05).

Variables	Mean rank		Mann-Whitney U	P value
	New survivor < 12 months of treatment completion months (N = 114)	Old survivors > 12 months of treatment completion (N = 54)		
Depression	86.69	66.4	1527.000	0.034
Anxiety	84	78.48	1889.500	0.557
Stress	84.25	77.38	1856.500	0.469
Body image score	88.53	58.1	1278.000	0.002

Breast Reconstruction Surgery

Breast reconstruction surgery can be beneficial towards the overall well-being of the woman post-mastectomy. Archangelo et al. (2019) performed a comparative, controlled study into the psychological effects of breast reconstruction post-mastectomy, whilst also looking at the contrast between mastectomy and non-mastectomy treatment patients [37]. The dependent variables included “sexual function, depression, and body image”, using once again the Beck Depression Inventory (BDI) mentioned previously, the Female Sexual Function Index (FSFI), and the Body Dysmorphic Disorder Examination (BDDE) to quantify the results [21,38,39]. The FSFI measures sexual function in women, with the version implemented in the study quoted using six domains. These include “desire, arousal, lubrication, orgasm, satisfaction and pain” [38]. Negative body image impression was evaluated with the BDDE, which incorporated 34 questions, and any score of 66 or above implied a degree of dissatisfaction or discomfort with self-body image. Results showed that for the patients included in the study between the ages of 18 and 67, those that had undergone a mastectomy had an FSFI score indicative of sexual dysfunction, compared to non-mastectomy patients, and this was corroborated by referenced studies. BDI scores were also higher in the mastectomy patients, with specific mention that changes in their physical appearance from these radical surgeries resulted in them feeling “less sexually attractive” and “less feminine”. The BDI results were not only categorised depending on treatment and reconstruction, but by age of the patients as well. It was shown that patients of a lower age (<47) in contribution with a history of depression, had a high predictor for future depression. Regarding the BDDE scores, the results were similar as with the FSFI and BDI indices. Patients who had undergone a form of mastectomy had higher BDDE scores or lower self-image perceptions in comparison with the control group. Overall, incorporating these three scoring methods, the authors were able to quantify the difference in sexual function, body image and depression in mastectomy patients and non-mastectomy patients [37]. 

Breast Conservation Therapy

Breast conservation therapy, also known as lumpectomy or wide local excision (WLE), can be considered by the surgeon when the stage of the tumour permits for them to have clear excisional margins, whilst maintaining a sufficient cosmetic outcome. Depending on the size and location of the tumour will determine whether breast-conserving surgery can be implemented or whether mastectomy would prove most beneficial [40]. When analysing these two groups of surgery, the complications associated with the surgery and radiation can determine a patient’s experience of the management. Admoun and Mayrovitz (2021) had 1606 breast cancer survivors respond to a questionnaire initially describing the surgery performed and any neo-adjuvant or adjuvant therapy (including breast reconstruction), then specify and quantify any complications that may have occurred [41]. These included any sensory changes, swelling, infections, necrosis, problems with implants, haematomas, ulcers, pain, and any cosmetical dissatisfaction. In the study, there were 978 mastectomy patients that responded to the questionnaire, and 628 lumpectomy patients. This allowed for a large sample size to gather results and further discuss the outcomes. As corroborated with previous studies, chronic pain post-surgery was the most frequently reported complication, with 72% of the total respondents having a degree of pain. Subdividing this, a greater proportion of these patients had undergone a lumpectomy, 78.8% relative to 64.7% [41]. Cosmetic satisfaction rates were also higher in lumpectomy patients. When evaluating an individual’s psychological and emotional outcome after breast surgery, a multivariable approach must be implemented. Boughton (2000) performed a meta-analysis of earlier studies on this topic, researching the importance of breast-conservation and the impact of age, on overall outcome [42]. Lumpectomies showed a benefit in regard to a woman’s “sense of attractiveness” and “feelings of attractiveness to their partner”. Emphasis was then placed on the importance of thoroughly preparing patients prior to any form of surgery that they would undergo. Knowledge surrounding the topic of emotional and psychological distress can improve self-management of the iatrogenic complications [42].

Limitations

Limitations of the studies analysed in the review were incorporated in respective sections. The majority of the studies used were survey-based, which can be subject to misunderstanding and variation of patient’s emotions and judgements on a day-to-day basis. Many of the studies included had small sample sizes which can decrease the reliability and generality of the results. This includes the study on psychological barriers to attending national screening. Similarly, the studies by Silva et al. (2022) and Sharma and Purkayastha (2017) reviewing radiotherapy side effects and the impact on quality of life, concentrated solely on post-mastectomy patients [23,24]. Expanding this over all breast cancer patients receiving radiotherapy would provide a greater population analysis. This limited sample size was a recurrent limitation among many of the studies included in this review. Attempt was made however to review meta-analyses to decrease the impact of this issue.

## Conclusions

Breast cancer affects every patient in different ways. Each patient will have their own timeline of various treatments, with diverse side effects negatively impacting their quality of life. Breast cancer screening is one of the three cancer screening programmes in the UK, and despite its profound rate of success in preventing higher grades and stages, it does have adverse effects on the individual. Changes have been implanted with certain rapid diagnostic clinics to tackle the issues analysed. To tackle this issue of psychological barriers, the National Health Service (NHS) and breast cancer charities must continue to raise awareness surrounding screening. Studying pre-diagnostic emotions allows healthcare professionals to account for patients' needs throughout their cancer journey. Individuals’ sentiments will constantly vary thereby emphasising the importance of incorporating the biopsychosocial model during different appointments. Healthcare professional education surrounding the topic of this holistic model could be promoted thereby increasing the emphasis on implementing and encouraging psychosocial services. Further along the cancer journey, the types of non-surgical treatments were covered, with analysis into the most frequent negative impacts on quality of life. Solutions have been researched to treat these side effects as well as prophylaxis prior to presentation. 

In regard to breast cancer care, there is a need to enhance our understanding of the mental health challenges that are faced by patients throughout their care. By identifying these issues frequently, we can develop comprehensive approaches to management for the patients and their support network. There is also an importance on further education of the healthcare professionals involved in the care of breast cancer patients during the different stages of their journey. Having a lower threshold for identification of possible diagnoses of these psychological signs and symptoms can lead to improved overall patient results. Finally, continued research amongst the medical and clinical psychology teams is of vital importance when trying to find different methods of diagnosis and further management of the conditions described in this review. Incorporating these will hopefully lead to an increase in quality of life and treatment for patients. Overall, the problems relating to the psychological impact of breast cancer on patients have been sufficiently identified over the years. A possible recommendation hereinafter lies within patient and healthcare professional education prior to screening, investigation and commencing treatment regarding what the future may hold. Support needs to be provided at each stage in a patient’s cancer journey to care for them whatever the outcome. Increasing patient education in the form of clearly understanding the process and pathway that healthcare professionals follow regarding breast cancer, as well as directing patients to reliable sources and possible support groups if required. Tracking progress during cancer diagnosis, treatment and remission can also help patients understand where they are currently on this pathway. Open communication must be at the forefront of both the patients and their relatives, as well as the minds of the healthcare professionals, as this allows for any necessary questions to be asked from both sides.

## References

[1] (2024). Breast cancer statistics. https://www.wcrf.org/cancer-trends/breast-cancer-statistics/.

[2] (2024). Breast cancer facts and statistics. https://breastcancernow.org/about-us/why-we-do-it/breast-cancer-facts-and-statistics/.

[3] Engel GL (1977). The need for a new medical model: a challenge for biomedicine. Science.

[4] Koo MM, von Wagner C, Abel GA, McPhail S, Rubin GP, Lyratzopoulos G (2017). Typical and atypical presenting symptoms of breast cancer and their associations with diagnostic intervals: evidence from a national audit of cancer diagnosis. Cancer Epidemiol.

[5] (2023). We’re breast cancer now. https://breastcancernow.org/.

[6] İzci F, İlgün AS, Fındıklı E, Özmen V (2016). Psychiatric symptoms and psychosocial problems in patients with breast cancer. J Breast Health.

[7] Advisory Committee on Breast Cancer Screening (2006). Screening for breast cancer in England: past and future. J Med Screen.

[8] (2024). Breast screening pathway requirements specification. https://www.gov.uk/government/publications/breast-screening-pathway-requirements-specification/breast-screening-pathway-requirements-specification.

[9] Lourenco AP, Baird GL (2017). Anxiety and breast imaging--can community education by a breast radiologist decrease anxiety and improve knowledge?. Breast J.

[10] Lee J, Hardesty LA, Kunzler NM, Rosenkrantz AB (2016). Direct interactive public education by breast radiologists about screening mammography: impact on anxiety and empowerment. J Am Coll Radiol.

[11] Barentsz MW, Wessels H, van Diest PJ (2014). Same-day diagnosis based on histology for women suspected of breast cancer: high diagnostic accuracy and favorable impact on the patient. PLoS One.

[12] Consedine NS, Magai C, Neugut AI (2004). The contribution of emotional characteristics to breast cancer screening among women from six ethnic groups. Prev Med.

[13] Sharif SP, Ahadzadeh AS, Ong FS, Naghavi N (2020). Fear of negative appearance evaluation and attitude towards mammography: Moderating role of internal health locus of control,cancer worry and age. Health Promot Perspect.

[14] Clark MA, Bonacore L, Wright SJ, Armstrong G, Rakowski W (2003). The cancer screening project for women: experiences of women who partner with women and women who partner with men. Women Health.

[15] Maheu C, Lord B, Wang C, Tanimizu A, McCready D, Galica J, Howell D (2022). Understanding women's anxiety and uncertainty attending a rapid diagnostic clinic for suspicious breast abnormality: a mixed methods study. McGill J Med.

[16] Zanuso V, Fregoni V, Gervaso L (2020). Side effects of adjuvant chemotherapy and their impact on outcome in elderly breast cancer patients: a cohort study. Future Sci OA.

[17] Hwang SY, Chang SJ, Park BW (2013). Does chemotherapy really affect the quality of life of women with breast cancer?. J Breast Cancer.

[18] (2023). Functional assessment of cancer therapy - breast. https://www.facit.org/measures/fact-b.

[19] Glatzer W, Gulyas J (2014). Cantril self-anchoring striving scale. Encyclopedia of Quality of Life and Well-Being Research.

[20] McElduff P, Boyes A, Zucca A, Girgis A (2004). Supportive care needs survey: a guide to administration, scoring and analysis. https://www.researchgate.net/publication/239565229_Supportive_Care_Needs_Survey_A_guide_to_administration_scoring_and_analysis.

[21] Jackson-Koku G (2016). Beck depression inventory. Occup Med (Lond).

[22] Luz FA, Marinho ED, Nascimento CP (2022). The effectiveness of radiotherapy in preventing disease recurrence after breast cancer surgery. Surg Oncol.

[23] Silva AS, França AC, Padilla MP, Macedo LS, Magliano CA, Santos MD (2022). Brazilian breast cancer patient-reported outcomes: What really matters for these women. Front Med Technol.

[24] Sharma N, Purkayastha A (2017). Impact of radiotherapy on psychological, financial, and sexual aspects in postmastectomy carcinoma breast patients: a prospective study and management. Asia Pac J Oncol Nurs.

[25] Aranda S, Schofield P, Weih L, Yates P, Milne D, Faulkner R, Voudouris N (2005). Mapping the quality of life and unmet needs of urban women with metastatic breast cancer. Eur J Cancer Care (Engl).

[26] Costa WA, Monteiro MN, Queiroz JF, Gonçalves AK (2017). Pain and quality of life in breast cancer patients. Clinics (Sao Paulo).

[27] Greco MT, Roberto A, Corli O, Deandrea S, Bandieri E, Cavuto S, Apolone G (2014). Quality of cancer pain management: an update of a systematic review of undertreatment of patients with cancer. J Clin Oncol.

[28] Cobo-Cuenca AI, Martín-Espinosa NM, Sampietro-Crespo A, Rodríguez-Borrego MA, Carmona-Torres JM (2018). Sexual dysfunction in Spanish women with breast cancer. PLoS One.

[29] Boswell EN, Dizon DS (2015). Breast cancer and sexual function. Transl Androl Urol.

[30] Boland V, Brady AM, Drury A (2020). The physical, psychological and social experiences of alopecia among women receiving chemotherapy: An integrative literature review. Eur J Oncol Nurs.

[31] Lemieux J, Maunsell E, Provencher L (2008). Chemotherapy-induced alopecia and effects on quality of life among women with breast cancer: a literature review. Psychooncology.

[32] Ikeda M, Tamai N, Kanai H (2020). Effects of the appearance care program for breast cancer patients receiving chemotherapy: a mixed method study. Cancer Rep (Hoboken).

[33] Cherny NI, Paluch-Shimon S, Berner-Wygoda Y (2018). Palliative care: needs of advanced breast cancer patients. Breast Cancer (Dove Med Press).

[34] Rodin G, Lo C, Rydall A (2018). Managing cancer and living meaningfully (CALM): a randomized controlled trial of a psychological intervention for patients with advanced cancer. J Clin Oncol.

[35] Thakur M, Sharma R, Mishra AK, Singh K, Kar SK (2022). Psychological distress and body image disturbances after modified radical mastectomy among breast cancer survivors: a cross-sectional study from a tertiary care centre in North India. Lancet Reg Health Southeast Asia.

[36] Hopwood P, Fletcher I, Lee A, Al Ghazal S (2001). A body image scale for use with cancer patients. Eur J Cancer.

[37] Archangelo SD, Sabino M Neto, Veiga DF, Garcia EB, Ferreira LM (2019). Sexuality, depression and body image after breast reconstruction. Clinics (Sao Paulo).

[38] Meston CM, Freihart BK, Handy AB, Kilimnik CD, Rosen RC (2020). Scoring and interpretation of the FSFI: what can be learned from 20 years of use?. J Sex Med.

[39] Rosen JC, Reiter J (1996). Development of the body dysmorphic disorder examination. Behav Res Ther.

[40] Golara A, Kozłowski M, Lubikowski J, Cymbaluk-Płoska A (2024). Types of breast cancer surgery and breast reconstruction. Cancers (Basel).

[41] Admoun C, Mayrovitz H (2021). Choosing mastectomy vs. lumpectomy-with-radiation: experiences of breast cancer survivors. Cureus.

[42] Boughton B (2000). Emotional outcome after breast surgery is highly individual. J Natl Cancer Inst.

